# Interleukin-8 Release Inhibitors Generated by Fermentation of *Artemisia princeps* Pampanini Herb Extract With *Lactobacillus plantarum* SN13T

**DOI:** 10.3389/fmicb.2020.01159

**Published:** 2020-06-03

**Authors:** Tomoko Okamoto, Sachiko Sugimoto, Masafumi Noda, Tomoharu Yokooji, Narandalai Danshiitsoodol, Fumiko Higashikawa, Masanori Sugiyama

**Affiliations:** ^1^Department of Probiotic Science for Preventive Medicine, Graduate School of Biomedical and Health Sciences, Hiroshima University, Hiroshima, Japan; ^2^Department of Frontier Science for Pharmacotherapy, Graduate School of Biomedical and Health Sciences, Hiroshima University, Hiroshima, Japan; ^3^Department of Pharmacognosy, Graduate School of Biomedical and Health Sciences, Hiroshima University, Hiroshima, Japan

**Keywords:** *Artemisia princeps* Pampanini, lactic acid bacteria, *Lactobacillus plantarum*, medicinal plant, sesquiterpene lactone

## Abstract

Some glycosides, which are detected in water extracts from medicinal plants, have been reported to be degraded into their aglycones by incubating with some microorganisms producing β-glucosidase. We have shown that a plant-derived *Lactobacillus plantarum* SN13T harbors 11 open reading frames (ORFs) encoding the β-glucosidase enzyme and can grow vigorously in several herbal water extracts. In this study, we observed that the water extract from *Artemisia princeps* Pampanini (AP) fermented with the SN13T strain strongly inhibited the release of interleukin (IL)-8 from the HuH-7 cells, when compared to that without fermentation. Furthermore, we demonstrated that the SN13T strain produced at least two bioactive compounds from some compounds contained in AP extract. In addition, we determined that the two compounds were catechol and seco-tanapartholide C, which dose-dependently inhibited the release of IL-8. Because some sesquiterpene lactones are useful in pharmaceuticals, seco-tanapartholide C may be useful as an anti-inflammatory agent. This study suggests that the fermentation of medicinal herbs with *Lb. plantarum* SN13T is a significant technique to obtain bioactive compounds having therapeutic potential.

## Introduction

Medicinal herbs are important plants that have molecules with therapeutic potential (Newman and Cragg, 2012; Atanasov et al., 2015); however, the amounts of bioactive compounds in the plants are often small (Zhou et al., 2014). This is because the plant secondary metabolites are stored as glycosides in original plants for improving water solubility and chemical stability of the aglycones (Lee and Paik, 2017). In general, glycosides are shown to have lower biological activity than corresponding aglycones due to their low bioavailability. In addition, the amounts of aglycones are quite lower than that of their glycosides. For the plant secondary metabolites, the conversion to an active form (aglycone) is important to show their biological activity.

Deglycosylation reaction can release aglycones from their glycosides and increases biological activities of plant metabolites (Michlmayr and Kneifel, 2014). β-Glucosidase (EC 3.2.1.21), which is classified into a glycoside hydrolase family, releases aglycone from the β-glycosyl precursor. The enzyme is known to be widely distributed among animals, plants, fungi, yeasts, and bacteria (Lee and Paik, 2017). It has been reported that glycosides are generally converted to biologically active substances by intestinal microorganisms after ingestion (Akao et al., 1994; Tsuchihashi et al., 2009; Di Cagno et al., 2010; Amaretti et al., 2015; Cheng et al., 2016). For example, sennoside from *Rheum palmatum* is converted to its aglycone, sennidin formed by β-glucosidase from an intestinal microorganism (Akao et al., 1994). The resulting sennidin is further converted to rhein anthrone (Akao et al., 1994). β-glucosidase from an intestinal *Bifidobacterium* sp. SEN strain, which can recognize sennoside as a substrate, has been reported (Yang et al., 1996a, b). Since other intestinal bacteria scarcely degrade sennoside (Akao et al., 1994), the substrate specificity of β-glucosidase for the glycoside hydrolysis of plant secondary metabolites might be important to generate aglycone from glycosides. In another example, daidzin (isoflavone glycoside) contained in soy milk is converted to equol (isoflavone aglycone) by intestinal microorganisms (Di Cagno et al., 2010).

Phenolic compounds are detected in plant-based foods and beverages. Some of those have been found to possess important biological activities, including antioxidant, anti-inflammatory, anti-carcinogenic, and anti-microbial activities. Those compounds consist of thousands of molecules presenting a phenol ring, such as flavonoids, phenolic acids, lignans, and stilbenes, which are widely distributed in plants (Valdés et al., 2015). The compounds comprise as non-absorbable precursors, glycosides in their original plants. Only aglycones and some intact glucosides can be absorbed into the body (Williamson and Manach, 2005).

Thus, intestinal microorganisms play a significant role to convert the plant metabolites to their biological active aglycones. The intestinal biotransformation includes deglucosylation, dehydroxylation, reduction, C-ring cleavage, and demethylation (Di Cagno et al., 2010). Especially, glycoside hydrolysis will be an important step in the intestinal biotransformation of several kinds of plants. Fermentation of medicinal herbs with some microorganisms having the β-glucosidase is a useful method for converting the bioactive compounds from their precursor (Sheih et al., 2011; Seong et al., 2017; Tan et al., 2017). Fermentation technology is an attractive approach to improve the biological activity of plant secondary metabolites quantitatively and qualitatively.

Lactic acid bacterium (LAB) strains are one of the main microorganisms that organize the intestinal microbiota. More than 1,000 strains of LAB that have been isolated from fruits, vegetables, flowers, and medicinal plants are stored in Sugiyama’s laboratory. In the previous study, we have also evaluated their health benefits and analyzed the whole genome sequence of several LAB strains (Yasutake et al., 2016; Noda et al., 2018, 2019). In the preliminary study, we observed that the banana leaf-derived *Lactobacillus plantarum* SN13T could grow vigorously in the herbal water extract, but animal-derived LAB, which are originated from raw and fermented milks, could not (data not shown). In this study, we verified a hypothesis that the plant-derived LAB may convert some compounds contained in medicinal herbs to bioactive substances.

Medicinal herbs used for chronic diseases in Japan have been found to contain anti-inflammatory substances. Non-alcoholic steatohepatitis (NASH) is a chronic inflammatory disease with lifestyle-related diseases such as obesity, metabolic syndrome, and diabetes. Although the pharmacotherapies approved for NASH have not been known until now, there is possibility that the medicinal herbs fermented with LAB may possess compounds useful for the treatment to NASH. The NASH model used in this study has already been reported by Chavez-Tapia et al. (2012). In this model, the administration of fatty acids (FAs) increased the release of interleukin (IL)-8 in human hepatoma cell line (HuH-7 cells). The IL-8 is a potent chemoattractant of neutrophils and has been reported to be increased in NASH patients (Hill et al., 1993; Kim et al., 2006). Infiltrated neutrophil is one of the hallmarks of NASH and aggravates the inflammation of liver. Therefore, IL-8 is an important factor involved in the development and progression of NASH (Bertola et al., 2013; Chang et al., 2015).

When the inhibitory activity of IL-8 release was evaluated in some medicinal herbs using *in vitro* NASH model, “*Artemisia princeps* Pampanini” (AP) water extract fermented with SN13T strain exhibited inhibitory activity of IL-8 release. The AP contains lots of constituents of phenolic compounds such as flavonoids (eupatilin and jaceosidin) (Kim et al., 2008; Min et al., 2009) and caffeoylquinic acids (Lee et al., 2011), which have a wide range of bioactivities such as anti-inflammatory activity. The genus *Artemisia* also contains sesquiterpene lactones which are often detected in Compositae (Chadwick et al., 2013). Moreover, yomogin, which is classified into sesquiterpene lactones, detected in AP has been reported to exhibit intense anti-inflammatory activity (Ivanescu et al., 2015).

In this study, we evaluated the IL-8 inhibitory effect of the AP extract fermented with a few plant-derived LAB strains including the SN13T strain as compared to AP extract without fermentation. In addition, we determined the chemical structure of anti-inflammatory substances detected in the water-extracted AP fermented with the SN13T strain.

## Materials and Methods

### Materials

***Artemisia princeps*** Pampanini, the dried herb known as “Gaiyo-matsu” in Japan, was purchased from Kojima Kampo Co., Ltd. (Osaka, Japan). The ELISA kit for evaluating the release of IL-8 was purchased from PeploTech Co., Ltd. (Rocky Hill, CT, United States). Palmitate, oleate, phosphate-buffered saline (PBS), 0.5% (**w/v**) Trypsin-5.3 mmol ethylenediaminetetraacetic acid (EDTA)-4Na solution, an antibiotic solution composed of penicillin and streptomycin, and Cell Counting Kit-8 were purchased from Wako Co., Ltd. (Osaka, Japan). De Man, Rogosa, and Sharpe (MRS) medium was purchased from Becton Dickinson and Company (Tokyo, Japan). All chemicals were of the highest purity available.

### Culture Condition of the LAB Strains

*Lactobacillus plantarum* SN13T, *Lb. plantarum* SN35N, and *Pediococcus pentosaceus* LP28 were used. MRS broth (Merck KGaA, Darmstadt, Germany) was used as a medium for precultivation. The bacterium was grown at 28°C overnight in the MRS broth. After cultivation, the bacterial cells were collected by centrifugation, resuspended by the sterilized 0.85% (*w/v*) NaCl solution.

### Fermentation of AP Extract by LAB

*Artemisia princeps* Pampanini powder (5 g) was suspended in 100 mL of distilled water and boiled for 30 min. After cooling to room temperature, the suspension was centrifuged at 3,000 × *g* for 15 min at 20°C. The resulting supernatant fluid was filtrated with a paper filter and adjusted to a pH of 6.0. The solution was treated for 10 min at 100°C for sterilization, followed by cooling to room temperature. The sterilized 0.85% (*w/v*) NaCl solution or the SN13T cells suspended in the sterilized 0.85% (*w/v*) NaCl solution were added (final 1% *v/v*) to the sterilized herbal water extracts. The solutions were incubated for 24 h at 28°C, followed by centrifugation at 3,000 × *g* for 30 min to remove the bacterial cells. The supernatant fluid was used for the IL-8 release assay.

### Extraction of Anti-inflammatory Substances With Organic Solvent

To select the appropriate organic solvent, the supernatant fluid from the AP extract without fermentation (u-AP) and the AP extract fermented with the SN13T strain (f-AP-13T) was adjusted to a pH of 7.5 and extracted three times with an equal volume of ethyl acetate. The ethyl acetate was removed using a rotary evaporator at 42°C. The resulting residues were added to the 90% (*v/v*) MeOH, followed by extraction with an equal volume of n-hexane three times. The water fraction was additionally extracted with an equal volume of n-butanol three times. These four fractions (hexane fraction, 90% (*v/v*) MeOH fraction, n-butanol fraction, and water fraction) were dried and dissolved at 10 mg/mL.

### IL-8 Release Assay

IL-8 release assay using a NASH model was done according to previous reports (Chavez-Tapia et al., 2012). Briefly, the HuH-7 cells provided by JCRB Cell Bank, Osaka, Japan, were used at a density of 10 × 10^4^ cells/well in a 24-well plate and cultured in Dulbecco’s modified Eagle’s medium (DMEM) (low glucose) containing 10% (*v/v*) fetal bovine serum, 2 mM L-glutamine, 100 U/mL penicillin, and 100 μg/mL streptomycin for 24 h in an atmosphere of 5% CO_2_ at 37°C. Palmitate and oleate dissolved in DMSO were added to the cell culture at 600 μM (molar ratio of 1:2); simultaneously, herbal samples were added. After 24 h incubation, the concentration of IL-8 released into the medium was determined using a human IL-8 ELISA kit in accordance with the manufacturer’s instructions.

### Cell Viability Assay

Cell viability was measured using the Cell Counting Kit-8 in accordance with the manufacturer’s protocol. Briefly, HuH-7 cells were seeded in a 24-well plate at a density of 10 × 10^4^ cells/well and cultured for 24 h. Then, cells were replaced with DMEM medium containing optimal concentrations of FAs and herbal samples and cultured for another 24 h. After cultivation, serum-free DMEM containing kit reagent (WST-8) was added to each well and incubated for 1 h. Then, absorbance was measured at 450 nm using a microplate reader.

### High Performance Liquid Chromatography (HPLC) Analysis of 90% (*v/v*) MeOH Extracts

The 90% (*v/v*) MeOH extract (10 mg/mL) from the AP extract incubated with or without the LAB strain was eluted using HPLC (JASCO system; JASCO Corporation) with an octadecylsilyl (ODS) C18 column (Hydrosphere C18, YMC, Kyoto, Japan; 5 μm, Φ = 6 mm, *L* = 250 mm) at 37°C. The column was equilibrated with water and eluted with the linear gradient method (0 to 100% acetonitrile) at a flow rate of 1.0 mL/min. Wavelengths of 210 nm were employed to detect elution of the sample. The injection volume was 10 μL.

### Identification of Anti-inflammatory Compounds

To isolate the anti-inflammatory compounds detected in the f-AP-13T, the 90% (*v/v*) MeOH extract (2.1 g) obtained from 185 g dried AP was continuously fractionated with silica gel open column chromatography, ODS open column chromatography, and HPLC. For silica gel (Merck KGaA) column chromatography, the column was eluted with solvent three times the column volume as follows: chloroform–MeOH–water elusion system [(100:0:0), (20:1:0), (10:1:0), (5:1:0), (7:3:1), (6:4:1), (0:100:0)]. A biologically active fraction was obtained from (20:1:0) elution. Chromatography with an ODS (Nacalai Tesque, Kyoto, Japan) column was used for the resulting fraction (20:1:0). The column was eluted with solvent three times the column volume as follows: MeOH–water elution system [(3:7), (4:6), (5:5), (6:4), (7:3), (8:2), (9:1), (10:0)]. Two active fractions were obtained from the 30% (*v/v*) MeOH elution: the fraction eluted in the first half (1-1) and the fraction eluted in the last half (1-2). Next, purification with an HPLC using an ODS column (Inertsil ODS-3, GL Science, Tokyo, Japan; 3 μm, Φ = 4.6 mm, *L* = 250 mm) was carried out. For the 1-1 fraction, the column was eluted as follows: a three-solvent system (MeOH: acetone: water = 1.5:0.8:7.7) at 0.8 mL/min, and the eluate was monitored with a refractive index monitor, RI-8020 (TOSOH, Yamaguchi, Japan). For the 1-2 fraction, the column was eluted with a three-solvent system (MeOH: acetone: water = 2:1:7). Due to the intense activity of two fractions, their chemical structures were determined. ^1^H-NMR and ^13^C-NMR spectra were taken on a JEOL JNM-LA500 spectrometer at 500 and 125 MHz, respectively. MS spectra were taken on Thermo Fisher Scientific LTQ Orbitrap XL (HR-ESI-MS) and JEOL JMS-T100GCV (GC-MS).

### Data Analysis

Data were displayed as the mean ± standard deviation (SD) (*n* = 3). The differences in mean values between the groups were determined using one-way ANOVA, followed by a *post hoc* Tukey’s test. *p* < 0.05 was considered statistically significant.

## Results

### Effect of Water-Extracted AP Fermented With Three LAB Strains on the Release of IL-8 From HuH-7 Cells

**FIGURE 1:**
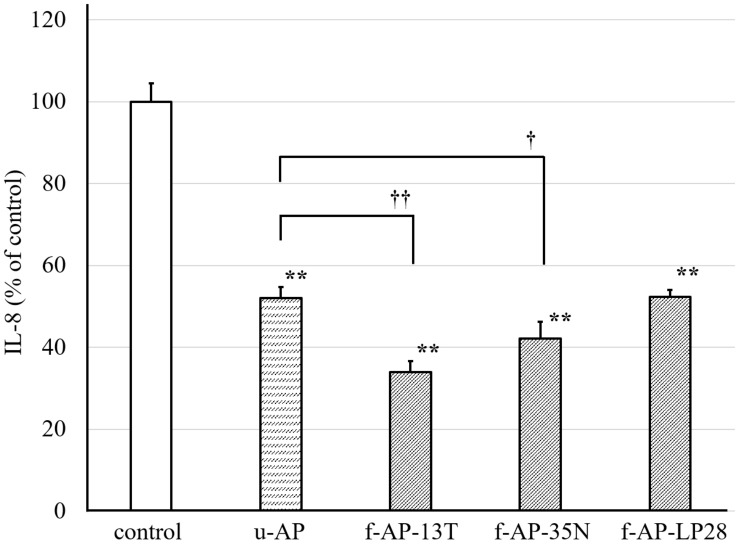
The inhibitory effect of the AP extract fermented with each LAB strain on the release of IL-8 from HuH-7 cells. In this experiment, *Lactobacillus plantarum* SN13T, *Lb. plantarum* SN35N, and *P. pentosaceus* LP28 were used. The AP extract was incubated for 24 h at 28°C with each strain, and the extract incubated without lactic acid bacterium was also prepared. The resulting sample was added to the HuH-7 cell culture medium at 45 μg/mL, along with FA treatment. The culture supernatant of the cells was collected 24 h after the treatment. The amount of IL-8 was measured by the ELISA method. Data are expressed as the mean ± standard deviation, (*n* = 3). **p < 0.01 versus control, ††*p* < 0.01 versus u-AP, †*p* < 0.05 versus u-AP. u-AP, AP extract without fermentation; f-AP-13T, AP extract fermented with the SN13T strain; f-AP-35N, AP extract fermented with the SN35N strain; f-AP-LP28, AP extract fermented with the LP28 strain.

With FA treatment, the amount of released IL-8 from HuH-7 was twice that of untreated cells (data not shown). We used three LAB strains, such as *Lb. plantarum* SN13T, pear-derived *Lb. plantarum* SN35N, and longan-derived *P. pentosaceus* LP28, which have 11, eight, and four open reading frames (ORFs) encoding β-glucosidase in their genome, respectively (Supplementary Figure S1). We measured the amount of IL-8 released from the HuH-7 cells treated with the water-extracted AP fermented with each LAB strain (Figure 1). The AP extract without fermentation (u-AP) decreased the amount of IL-8 released from the FA-treated HuH-7 cells to 50%. The AP extract fermented with the SN13T strain (f-AP-13T) or the SN35N strain (f-AP-35N) decreased the amount of IL-8 as compared to that without fermentation. However, the inhibitory effect of f-AP-13T was higher than that of f-AP-35N. Conversely, the AP extract fermented with the LP28 strain (f-AP-LP28) was almost the same as that without fermentation, suggesting that *Lb. plantarum* SN13T was the best of the three strains for fermenting water-extracted AP. The cell viability assay indicated that the concentration of herbal extracts and FAs used in this experiment do not exhibit cytotoxicity (data not shown).

### Extraction of Anti-inflammatory Substances From Fermented AP Extract

To identify chemically the anti-inflammatory substances in the AP extract fermented with the SN13T strain, the AP extract incubated with or without the SN13T strain was extracted with several organic solvents. After each water extract was fractionated with ethyl acetate, the ethyl acetate was removed by evaporation, and the residues were fractionated with hexane and 90% (*v/v*) MeOH. On the other hand, the water layer was extracted with n-butanol.

**FIGURE 2:**
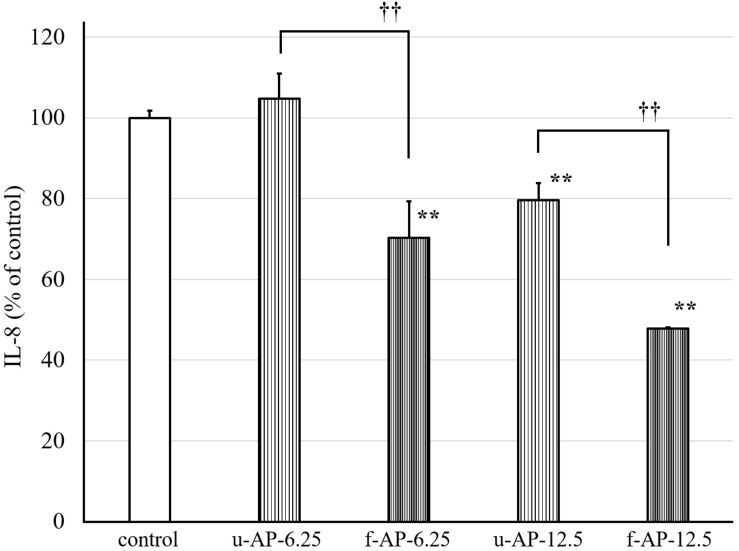
The effect of the 90% (*v/v*) MeOH extract on the release of IL-8 from HuH-7 cells. The AP extract, which was incubated with or without the SN13T strain, was extracted with some organic solvent. The 90% (*v/v*) MeOH extract was diluted and added to the HuH-7 cell culture at 6.25 or 12.5 μg/mL. The culture supernatant was obtained after 24 h cultivation to measure the amount of IL-8 by the ELISA method. Data are expressed as the mean ± standard deviation, (*n* = 3). **p < 0.01 versus control, ^††^*p* < 0.01 versus u-AP-6.25/u-AP-12.5. u-AP-6.25, 90% (*v/v*) MeOH extract from u-AP-13T (6.25 μg/mL); f-AP-6.25, 90% (*v/v*) MeOH extract from f-AP-13T (6.25 μg/mL); u-AP-12.5, 90% (*v/v*) MeOH extract from u-AP-13T (12.5 μg/mL); f-AP-12.5, 90% (*v/v*) MeOH extract from f-AP-13T (12.5 μg/mL).

The anti-inflammatory activity of four kinds of extracts [n-hexane fractions, 90% (*v/v*) MeOH fractions, n-butanol fractions, and water fractions] was evaluated by measuring the amount of IL-8 released from HuH-7 cells. The 90% (*v/v*) MeOH extract obtained from the ethyl acetate extract from the AP extract incubated with or without the SN13T strain dose-dependently inhibited the release of IL-8 from HuH-7 cells (Figure 2), whereas other extracts did not inhibit remarkably (data not shown). In addition, the 90% (*v/v*) MeOH extract from the AP extract fermented with the SN13T strain more clearly inhibited than that without fermentation.

### Constituents Analysis of the 90% (*v/v*) MeOH Extract

**FIGURE 3:**
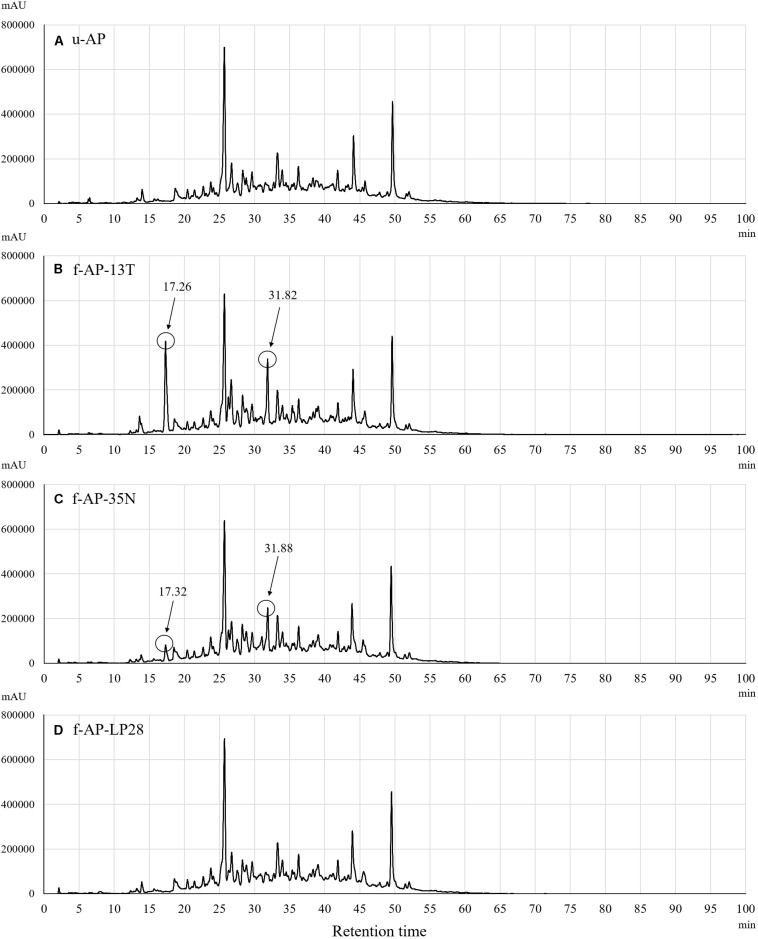
Constituent analysis in the 90% (*v/v*) MeOH extracts from the u-AP **(A)**, f-AP-13T **(B)**, f-AP-35N **(C)**, f-AP-LP28 **(D)**. HPLC was done under the conditions described in the section “Materials and Methods.” u-AP, 90% (*v/v*) MeOH extract from AP extract without fermentation; f-AP-13T, 90% (*v/v*) MeOH extract from AP extract fermented with the SN13T strain; f-AP-35N, 90% (*v/v*) MeOH extract from AP extract fermented with the SN35N strain; f-AP-LP28, 90% (*v/v*) MeOH extract from AP extract fermented with the LP28 strain.

To analyze the constituents of each MeOH extract, we compared the eluted profile of HPLC between the 90% (*v/v*) MeOH extract from u-AP and that from f-AP-13T. Figure 3 shows that two peaks are present at 17 and 32 min in f-AP-13T, whereas these peaks were not or only slightly observed in u-AP. Thus, we hypothesized that these two compounds might be mainly generated by fermentation and inhibited the release of IL-8 from FA-treated HuH-7 cells. Furthermore, the analysis was performed for the 90% (*v/v*) MeOH extracts from f-AP-35N and f-AP-LP28. In f-AP-35N, two peaks (17 and 32 min) were observed, but smaller than that of f-AP-13T. On the other hand, two peaks were scarcely observed in f-AP-LP28. These results suggest that the enhancement of the activity may be the compounds derived from these two peaks because of correlation between the productivity of these compounds and the activity level. These peak areas were as follows: peak area of the MeOH extract of u-AP (17.027 min–303597 uAU) (31.893 min–1322832 uAU); peak area of the MeOH extract of f-AP-13T (17.267 min–9014213 uAU) (31.827 min–7444605 uAU); peak area of the MeOH extract of f-AP-35N, (17.320 min–2132734 uAU) (31.880 min–6289482 uAU); and peak area of the MeOH extract of f-AP-LP28 (17.387 min–345139 uAU) (31.587 min–4004585 uAU).

### Identification of Anti-inflammatory Compounds in AP Extract Fermented With the SN13T Strain

**FIGURE 4:**
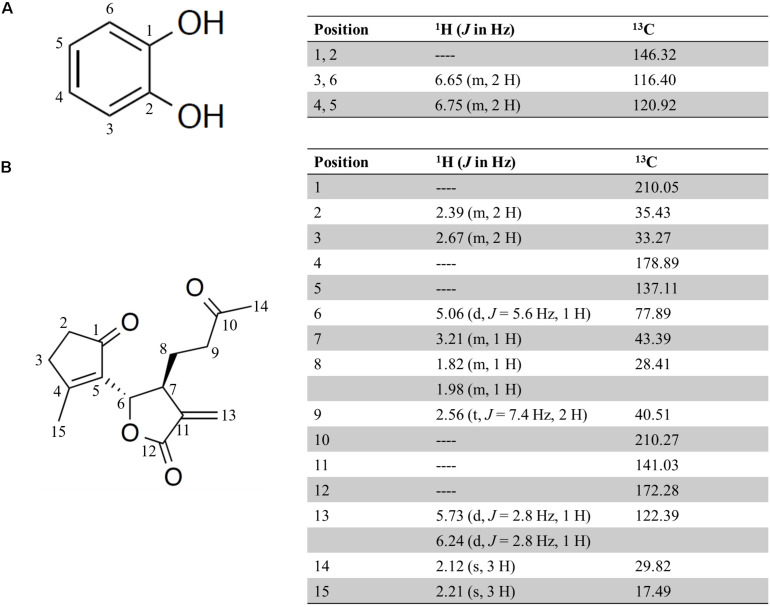
Chemical structure and ^1^H and ^13^C NMR data (500 and 125 MHz, Methanol-*d*_4_) for catechol **(A)** and seco-tanapartholide C **(B)**.

To identify the anti-inflammatory compounds contained in the 90% (*v/v*) MeOH extract from the AP extract fermented with the SN13T strain, further purification was done using a silica gel and octadecylsilanized silica gel column chromatography, and HPLC. After purification by HPLC using an ODS-3 C18 column, we obtained two active compounds that inhibit the release of IL-8 from HuH-7 cells. To determine these chemical structures, ^1^H-NMR, ^13^C-NMR, and high-resolution electrospray ionization mass spectrometry (HR-ESI-MS)/gas chromatography electron ionization mass spectrometry (GC-EI-MS) were used. The MS spectrum of each compound showed an ion at m/z 110.0 and 285.11, suggesting that the molecular formula of former and latter is C_6_H_6_O_2_ [M] and C_15_H_18_O_4_Na [M + Na], respectively. As a result, the two compounds were identified as catechol and seco-tanapartholide C, respectively, judging from the known analytical data (Liang et al., 2016; Zan et al., 2010). The chemical structures and spectral data of catechol and seco-tanapartholide C are shown in Figure 4.

### Inhibitory Effect of the Release of IL-8 in HuH-7 Cells by Catechol and Seco-Tanapartholide C

**FIGURE 5:**
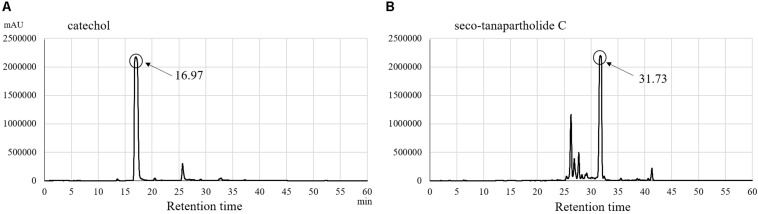
HPLC chromatograms of catechol **(A)** and seco-tanapartholide C **(B)**.

**FIGURE 6:**
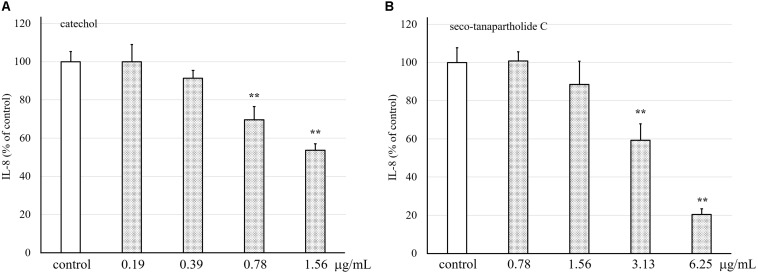
Effects of catechol and seco-tanapartholide C on the release of IL-8 from HuH-7 cells. Catechol **(A)** was used between 0.19 and 1.56 μg/mL. Seco-tanapartholide C **(B)** was used between 0.78 and 6.25 μg/mL. The supernatant from the HuH-7 cell culture harvested after 24 h was used to measure the amount of IL-8 by ELISA. Data were expressed as the mean ± standard deviation, (*n* = 3), **p < 0.01 versus control.

Purified catechol and seco-tanapartholide C, which were found in the 90% (*v/v*) MeOH extract, displayed the same retention times (17 and 32 min, respectively) as the authentic ones (Figure 5). In addition, we evaluated whether catechol and seco-tanapartholide C inhibit the release of IL-8 from HuH-7 cells. Both compounds dose-dependently inhibited the release of IL-8 (Figure 6). However, the peaks observed at retention times of 25–30 min in Figure 6B did not exhibit inhibitory activity (data not shown). These compounds were cytotoxic at higher concentrations than those used for the assay and could not be verified at higher concentrations. Finally, 22.3 and 18.0 mg of catechol and seco-tanapartholide C, respectively, were obtained from 185 g of the dried AP.

## Discussion

The biosynthesized plant secondary metabolites are stored as glycosides in the plant cells (Lee and Paik, 2017). Glycosides are less hydrophobic and less biologically active than their aglycones. Water-soluble extracts from medicinal plants contain large amounts of masked active compounds such as glycosides. It has been reported that glycosides are converted to aglycones by intestinal microorganisms after ingestion (Akao et al., 1994; Tsuchihashi et al., 2009; Di Cagno et al., 2010; Amaretti et al., 2015; Cheng et al., 2016). The fermentation of plants with microorganisms having β-glucosidase increases the amounts of bioactive compounds (Sheih et al., 2011; Seong et al., 2017; Tan et al., 2017). Therefore, fermentation techniques can be useful for generating active compounds from glycosides.

In our preliminary study, we observed that AP extract fermented with the SN13T strain inhibited the release of IL-8 from the HuH-7 cells. Furthermore, we analyzed the whole genome sequence of a few plant-derived LAB strains including *Lb. plantarum* SN13T to find the β-glucosidase-encoding gene in their genomes. In this study, we evaluated the IL-8 inhibitory effect of AP extract fermented with a few LAB strains, including the SN13T strain, and compared them to those of the AP extract without fermentation.

*In vitro* NASH models have been established using hepatoma cell lines, such as HepG2 and HuH-7, to screen the compounds with medicinal potential (Grasselli et al., 2017). In this study, we used HuH-7 cells treated with FAs as an *in vitro* NASH model established by Chavez-Tapia et al. (2012). In our experiment, the amount of IL-8 released into the medium was increased by two folds, whereas the amounts of TNF-α and IL-6 were scarcely detected due to detection limit of the ELISA (data not shown). In the previous experiment, it has been reported that the release of IL-8 from human primary hepatocytes and hepatoma cell line has been increased by the FA administration (Joshi-Barve et al., 2007). This suggests that this model used in this study may be suitable for evaluating the anti-inflammatory activity of water-extracted AP fermented with the LAB strains.

As shown in Figure 1, the anti-inflammatory activity of AP extract fermented with the SN13T or SN35N strain was higher than that without fermentation. In contrast, the anti-inflammatory activity of AP extract fermented with the LP28 strain was almost the same as that without fermentation, showing that the SN13T strain is suitable to obtain the bioactive substances generated by fermenting the AP extract. On the other hand, SN35N strain, which also harbors several β-glucosidase genes, was found to produce slight amount of the substances under the tested condition. Therefore, the culture condition may also affect the production of the compounds. We successfully purified catechol and seco-tanapartholide C exhibiting dose-dependently anti-inflammatory activity from the water-extracted AP fermented with SN13T strain (Figure 6). Since both compounds were scarcely detected in that without fermentation (Figure 3A), these compounds were mainly generated by fermentation.

The catechol and seco-tanapartholide C were slightly or scarcely detected in the AP extract fermented with the SN35N or LP28 strain, respectively (Figures 3C,D). Our preliminary analysis has suggested that the SN13T, SN35N, and LP28 strains harbor 11, eight, and four ORFs encoding β-glucosidase, respectively (Supplementary Figure S1). When a phylogenetic tree was constructed based on the amino acid sequence predicted from these putative β-glucosidases, the SN13T strain was shown to possess various β-glucosidases (Supplementary Figure S2). On the other hand, although almost β-glucosidases found in the LP28 strain are similar with some of those in the SN13T or SN35N strain, both SN13T and SN35N strains also harbor β-glucosidases which are not detected in the LP28 strain. Judging from these data, it is suggested that β-glucosidases uniquely found in the SN13T and SN35N strains may be able to recognize a plant secondary metabolite from *Artemisia princeps* Pampanini herb extract as a substrate to produce bioactive compounds. Therefore, it was suggested that microorganisms with various β-glucosidase, such as the SN13T strain, may be suitable for fermentation of medicinal herbs. However, the substrate specificity of β-glucosidase is somewhat broad, and the putative β-glucosidase-encoding genes appear redundant in the LAB genome (Michlmayr and Kneifel, 2014). Therefore, further study is needed to investigate β-glucosidase activity on the abundant and diverse plant secondary metabolites.

Catechol is classified into phenolics, which is abundantly present in Compositae (Heinrich et al., 1998). The common metabolites from flavonoid and non-flavonoid phenolics by microbial degradation in the human intestine are benzoic acid derivatives (C_6_-C_1_ skeleton), such as gallic acid, *p*-hydroxybenzoic acid, vanillic acid, protocatechuic acid (3,4-dihydroxybenzoic), and syringic acid. In these compounds, the protocatechuic acid is further converted into catechol (Selma et al., 2009). Additionally, in our previous study, the SN13T strain has been found to produce the catechol, when the strain was cultured in some plant-derived extracts (manuscript in preparation). In fact, there are some reports that catechol is generated by the fermentation of fruits and crops with *Lb. plantarum* (Ricci et al., 2019; Ryu et al., 2019; Xu et al., 2019). The strain may seem to produce the catechol through intermediates, 3-dehydroquinic acid, 3-dehydroshikimic acid, and protocatechuic acid from quinic acid and shikimic acid via shikimate pathway (Ghosh et al., 2012).

Phenols are effective antioxidants that may protect against several chronic inflammatory diseases (Pandey and Rizvi, 2009). Catechol also has anti-inflammatory activity, and its mechanisms are partially understood. The catechol not only directly scavenges NO radicals but also down-regulates the iNOS expression via the inhibition of NF-κB resulting in the reduction of NO production (Kazłowska et al., 2010; Fernando et al., 2016). Catechol may suppress the production of IL-8 resulting from the inhibition of the NF-κB signaling pathway.

Seco-tanapartholide C is classified in guaiane-type sesquiterpene lactones (SQLs), which are also abundantly present in Compositae (Heinrich et al., 1998). Guaiane-type SQLs have also been found in the glucoside form (Fontanel et al., 1999). Therefore, seco-tanapartholide C may be generated by catalytic activity of β-glucosidase in the fermentation process using the SN13T strain. However, since seco-tanapartholide C does not harbor a hydroxyl group bound to sugar, a further reaction, such as dehydroxylation, may be occurred to generate seco-tanapartholide C after hydrolysis with β-glucosidase. Dehydroxylation reaction is generally observed among intestinal microorganisms (Di Cagno et al., 2010), and thus, the reaction may be progressed during the fermentation with SN13T strain.

A previous report has shown that guaiane-type SQL have a wide range of pharmacological activities including anticancer, anti-inflammatory, antioxidant, and antibacterial effects (Liu et al., 2018). Artemisinin, which is a well-known antimalarial drug, is also classified into SQL and is in clinical trials as an anti-cancer drug (Ghantous et al., 2010). Thus, SQL is important as a lead compound for drug discovery. Seco-tanapartholide C belongs to 1,10-seco-guaianolides which show anti-inflammatory effects due to the inhibition of the NF-κB signaling pathway (Makiyi et al., 2009; Liu et al., 2018). Joshi-Barve et al. (2007) have reported that IL-8 expression is mediated by NF-κB in the *in vitro* NASH cell model. The pharmacological activity of SQL has been reportedly ascribed to the alkylation of thiol groups in target proteins by the α, β-unsaturated carbonyl group in its structure (Chadwick et al., 2013). Since seco-tanapartholide C possesses two α, β-unsaturated carbonyl groups in its structure, the compound may suppress the production of IL-8 resulting from the inhibition of the NF-κB signaling pathway. NF-κB, which is known as a ubiquitous protein, regulates over 150 inflammatory genes (such as cytokines, inflammatory molecules, and cell adhesion molecules) and mediates immune response in humans (Lee et al., 2011). Therefore, inhibition of NF-κB is useful to decrease inflammatory response and suppresses cancer growth. Seco-tanapartholide C may be useful as an anti-inflammatory agent.

In this study, we found that catechol and seco-tanapartholide C were newly produced in the AP extract fermented with *Lb. plantarum* SN13T.

## Conclusion

The AP extract fermented with the SN13T strain increased the amounts of the catechol and seco-tanapartholide C by converting their precursor. This suggests that the fermentation of a medicinal herb with the *Lb. plantarum* SN13T is a significant technique for obtaining active compounds with therapeutic potential.

## Data Availability Statement

All datasets generated for this study are included in the article/Supplementary Material.

## Author Contributions

TO and MS were responsible for the conceptualization of the study and contributed to project administration. TO, SS, MN, and ND contributed to the methodology. TO was responsible for the formal analysis of the study and visualization of the study. TO and SS were responsible for the investigation of the study. MS contributed to the resources and responsible for funding acquisition. TO, MN, TY, FH, and MS were responsible for the original draft preparation. TO, MN, and MS were responsible for review and editing of the manuscript. All authors have read and agreed to the published version of the manuscript.

## Conflict of Interest

The authors declare that the research was conducted in the absence of any commercial or financial relationships that could be construed as a potential conflict of interest.

## References

[B1] Akao T. Che Q. M. Kobashi K. Yang L. Hattori M. Namba T. (1994). Isolation of a human intestinal anaerobe, *Bifidobacterium* sp. strain SEN, capable of hydrolyzing sennosides to sennidins. *Appl. Environ. Microbiol.* 60 1041–1043. 8161172 10.1128/aem.60.3.1041-1043.1994 PMC201432

[B2] Amaretti A. Raimondi S. Leonardi A. Quartieri A. Rossi M. (2015). Hydrolysis of the rutinose-conjugates flavonoids rutin and hesperidin by the gut microbiota and bifidobacteria. *Nutrients* 7 2788–2800. 10.3390/nu7042788 25875120 PMC4425173

[B3] Atanasov A. G. Waltenberger B. Pferschy-Wenzig E. M. Linder T. Wawrosch C. Uhrin P. (2015). Discovery and resupply of pharmacologically active plant-derived natural products: a review. *Biotechnol. Adv.* 33 1582–1614. 10.1016/j.biotechadv.2015.08.001 26281720 PMC4748402

[B4] Bertola A. Park O. Gao B. (2013). Chronic plus binge ethanol feeding synergistically induces neutrophil infiltration and liver injury in mice: a critical role for E-selectin. *Hepatology* 58 1814–1823. 10.1002/hep.26419 23532958 PMC3726575

[B5] Chadwick M. Trewin H. Gawthrop F. Wagstaff C. (2013). Sesquiterpenoids lactones: benefits to plants and people. *Int. J. Mol. Sci.* 14 12780–12805. 10.3390/ijms140612780 23783276 PMC3709812

[B6] Chang B. Xu M. J. Zhou Z. Cai Y. Li M. Wang W. (2015). Short- or long-term high-fat diet feeding plus acute ethanol binge synergistically induce acute liver injury in mice: an important role for CXCL1. *Hepatology* 62 1070–1085. 10.1002/hep.27921 26033752 PMC4589443

[B7] Chavez-Tapia N. C. Rosso N. Tiribelli C. (2012). Effect of intracellular lipid accumulation in a new model of non-alcoholic fatty liver disease. *BMC Gastroenterol.* 12:20. 10.1186/1471-230X-12-20 22380754 PMC3313845

[B8] Cheng J. R. Liu X. M. Chen Z. Y. Zhang Y. S. Zhang Y. H. (2016). Mulberry anthocyanin biotransformation by intestinal probiotics. *Food Chem.* 213 721–727. 10.1016/j.foodchem.2016.07.032 27451240

[B9] Di Cagno R. Mazzacane F. Rizzello C. G. Vincentini O. Silano M. Giuliani G. (2010). Synthesis of isoflavone aglycones and equol in soy milks fermented by food-related lactic acid bacteria and their effect on human intestinal caco-2 cells. *J. Agric. Food Chem.* 58 10338–10346. 10.1021/jf101513r 20822177

[B10] Fernando I. P. S. Nah J. W. Jeon Y. J. (2016). Potential anti-inflammatory natural products from marine algae. *Environ. Toxicol. Pharmacol.* 48 22–30. 10.1016/j.etap.2016.09.023 27716532

[B11] Fontanel D. Galtier C. Debouzy J. C. Gueiffier A. Viel C. (1999). Sesquiterpene lactone glycosides from *Lapsana communis* L. subsp. *communis*. *Phytochemistry* 51 999–1004. 10444857 10.1016/s0031-9422(98)00718-3

[B12] Ghantous A. Gali-Muhtasib H. Vuorela H. Saliba N. A. Darwiche N. (2010). What made sesquiterpene lactones reach cancer clinical trials? *Drug Discov. Today* 15 668–678. 10.1016/j.drudis.2010.06.002 20541036

[B13] Ghosh S. Chisti Y. Banerjee U. C. (2012). Production of shikimic acid. *Biotechnol. Adv.* 30 1425–1431. 10.1016/j.biotechadv.2012.03.001 22445787

[B14] Grasselli E. Canesi L. Portincasa P. Voci A. Vergani L. Demori I. (2017). Models of non-alcoholic fatty liver disease and potential translational value: the effects of 3,5-L-diiodothyronine. *Ann. Hepatol.* 16 707–719. 10.5604/01.3001.0010.2713 28809727

[B15] Heinrich M. Robles M. West J. E. Ortiz De Montellano B. R. Rodriguez E. (1998). Ethnopharmacology of Mexican asteraceae (compositae). *Annu. Rev. Pharmacol. Toxicol.* 38 539–565. 9597165 10.1146/annurev.pharmtox.38.1.539

[B16] Hill D. L. B. Marsano L. S. McClain C. J. (1993). Increased plasma interleukin−8 concentrations in alcoholic hepatitis. *Hepatology* 18 576–580. 8359798

[B17] Ivanescu B. Miron A. Corciova A. (2015). Sesquiterpene lactones from *Artemisia* Genus: biological activities and methods of analysis. *J. Anal. Methods Chem.* 2015:247685. 10.1155/2015/247685 26495156 PMC4606394

[B18] Joshi-Barve S. Barve S. S. Amancherla K. Gobejishvili L. Hill D. Cave M. (2007). Palmitic acid induces production of proinflammatory cytokine interleukin-8 from hepatocytes. *Hepatology* 46 823–830. 17680645 10.1002/hep.21752

[B19] Kazłowska K. Hsu T. Hou C. C. Yang W. C. Tsai G. J. (2010). Anti-inflammatory properties of phenolic compounds and crude extract from *Porphyra dentata*. *J. Ethnopharmacol.* 128 123–130. 10.1016/j.jep.2009.12.037 20051261

[B20] Kim C. S. Park H. S. Kawada T. Kim J. H. Lim D. Hubbard N. E. (2006). Circulating levels of MCP-1 and IL-8 are elevated in human obese subjects and associated with obesity-related parameters. *Int. J. Obes.* 30 1347–1355. 16534530 10.1038/sj.ijo.0803259

[B21] Kim M. J. Han J. M. Jin Y. Y. Baek N. Bang M. H. Chung H. G. (2008). In vitro antioxidant and anti-inflammatory activities of jaceosidin from *Artemisia princeps* Pampanini cv. Sajabal. *Arch. Pharm. Res.* 31 429–437. 10.1007/s12272-001-1175-8 18449499

[B22] Lee N. K. Paik H. D. (2017). Bioconversion using lactic acid bacteria: ginsenosides, gaba, and phenolic compounds. *J. Microbiol. Biotechnol.* 27 869–877. 10.4014/jmb.1612.12005 28297748

[B23] Lee S. G. Lee H. Nam T. G. Eom S. H. Heo H. J. Lee C. Y. (2011). Neuroprotective effect of caffeoylquinic acids from *Artemisia princeps* Pampanini against oxidative stress-induced toxicity in PC-12 Cells. *J. Food Sci.* 76 250–256. 10.1111/j.1750-3841.2010.02010.x 21535743

[B24] Liang Y. F. Li X. Wang X. Zou M. Tang C. Liang Y. (2016). Conversion of simple cyclohexanones into catechols. *J. Am. Chem. Soc.* 138 12271–12277. 10.1021/jacs.6b07269 27564642

[B25] Liu L. Dai W. Xiang C. Chi J. Zhang M. (2018). 1,10-Secoguaianolides From *Artemisia Austro-Yunnanensis* and their anti-inflammatory effects. *Molecules* 23 1–14. 10.3390/molecules23071639 29976846 PMC6099792

[B26] Makiyi E. F. Frade R. F. Lebl T. Jaffray E. G. Cobb S. E. Harvey A. L. (2009). Iso-seco-tanapartholides: isolation, synthesis and biological evaluation. *Eur. J. Org. Chem.* 2009 5711–5715. 23606807 10.1002/ejoc.200901016 PMC3627315

[B27] Michlmayr H. Kneifel W. (2014). β-Glucosidase activities of lactic acid bacteria: mechanisms, impact on fermented food and human health. *FEMS Microbiol. Lett.* 352 1–10. 10.1111/1574-6968.12348 24330034

[B28] Min S. W. Kim N. J. Baek N. I. Kim D. H. (2009). Inhibitory effect of eupatilin and jaceosidin isolated from *Artemisia princeps* on carrageenan-induced inflammation in mice. *J. Ethnopharmacol.* 125 497–500. 10.1016/j.jep.2009.06.001 19505561

[B29] Newman D. J. Cragg G. M. (2012). Natural products as sources of new drugs over the 30 Years from 1981 to 2010. *J. Nat. Prod.* 75 311–335. 10.1021/np200906s 22316239 PMC3721181

[B30] Noda M. Danshiitsoodol N. Inoue Y. Okamoto T. Sultana N. Sugiyama M. (2019). Antibiotic susceptibility of plant-derived lactic acid bacteria conferring health benefits to human. *J. Antibiot.* 72 834–842. 10.1038/s41429-019-0218-4 31399643

[B31] Noda M. Shiraga M. Kumagai T. Danshiitsoodol N. Sugiyama M. (2018). Characterization of the SN35N strain-specific exopolysaccharide encoded in the whole circular genome of a plant-derived *Lactobacillus plantarum*. *Biol. Pharm. Bull.* 41 536–545. 10.1248/bpb.b17-00840 29607926

[B32] Pandey K. B. Rizvi S. I. (2009). Plant polyphenols as dietary antioxidants in human health and disease. *Oxid Med Cell Longev.* 2 270–278. 10.4161/oxim.2.5.9498 20716914 PMC2835915

[B33] Ricci A. Cirlini M. Calani L. Bernini V. Neviani E. Del Rio D. (2019). *In vitro* metabolism of elderberry juice polyphenols by lactic acid bacteria. *Food Chem.* 276 692–699. 10.1016/j.foodchem.2018.10.046 30409649

[B34] Ryu J. Y. Kang H. R. Cho S. K. (2019). Changes over the fermentation period in phenolic compounds and antioxidant and anticancer activities of blueberries fermented by *Lactobacillus plantarum*. *J. Food. Sci.* 84 2347–2356. 10.1111/1750-3841.14731 31313311

[B35] Selma M. V. Espín J. C. Tomás-Barberán F. A. (2009). Interaction between phenolics and gut microbiota: role in human health. *J. Agric. Food Chem.* 57 6485–6501. 10.1021/jf902107d 19580283

[B36] Seong J. S. Xuan S. H. Park S. H. Lee K. S. Park Y. M. Park S. N. (2017). Antioxidative and antiaging activities and component analysis of *Lespedeza cuneata* G. Don extracts fermented with *Lactobacillus pentosus*. *J. Microbiol. Biotechnol.* 27 1961–1970. 10.4014/jmb.1706.06028 28910861

[B37] Sheih I. C. Fang T. J. Wu T. K. Chang C. H. Chen R. Y. (2011). Purification and properties of a novel phenolic antioxidant from *Radix astragali* fermented by *Aspergillus oryzae* M29. *J. Agric. Food Chem.* 59 6520–6525. 10.1021/jf2011547 21557623

[B38] Tan J. S. Yeo C. R. Popovich D. G. (2017). Fermentation of protopanaxadiol type ginsenosides (PD) with probiotic *Bifidobacterium lactis* and *Lactobacillus rhamnosus*. *Appl. Microbiol. Biotechnol.* 101 5427–5437. 10.1007/s00253-017-8295-4 28478490

[B39] Tsuchihashi R. Kodera M. Sakamoto S. Nakajima Y. Yamazaki T. Niiho Y. (2009). Microbial transformation and bioactivation of isoflavones from Pueraria flowers by human intestinal bacterial strains. *J. Nat. Med.* 63 254–260. 10.1007/s11418-009-0322-z 19219523

[B40] Valdés L. Cuervo A. Salazar N. Ruas-Madiedo P. Gueimondea M. González S. (2015). The relationship between phenolic compounds from diet and microbiota: impact on human health. *Food Funct.* 6 2424–2439. 10.1039/c5fo00322a 26068710

[B41] Williamson G. Manach C. (2005). Bioavailability and bioefficacy of polyphenols in humans. II. Review of 93 intervention studies. *Am. J. Clin. Nutr.* 81 243S–255S. 10.1093/ajcn/81.1.243S 15640487

[B42] Xu D. Ding W. Ke W. Li F. Zhang P. Guo X. (2019). Modulation of metabolome and bacterial community in whole crop corn silage by inoculating homofermentative *Lactobacillus plantarum* and heterofermentative *Lactobacillus buchneri*. *Front. Microbiol.* 9:3299. 10.3389/fmicb.2018.03299 30728817 PMC6352740

[B43] Yang L. Akao T. Kobashi K. Hattori M. (1996a). A sennoside-hydrolyzing beta-glucosidase from *Bifidobacterium* sp. strain SEN is inducible. *Biol. Pharm. Bull.* 19 701–704. 8741578 10.1248/bpb.19.701

[B44] Yang L. Akao T. Kobashi K. Hattori M. (1996b). Purification and characterization of a novel sennoside-hydrolyzing beta-glucosidase from *Bifidobacterium* sp. strain SEN, a human intestinal anaerobe. *Biol. Pharm. Bull.* 19 705–709. 8741579 10.1248/bpb.19.705

[B45] Yasutake T. Kumagai T. Inoue A. Kobayashi K. Noda M. Orikawa A. (2016). Characterization of the LP28 strain-specific exopolysaccharide biosynthetic gene cluster found in the whole circular genome of *Pediococcus pentosaceus*. *Biochem. Biophys. Rep.* 5 266–271. 10.1016/j.bbrep.2016.01.004 28955833 PMC5600453

[B46] Zan K. Chen X. Q. Fu Q. Shi S. P. Zhou S. X. Xiao M. T. (2010). 1, 10-Secoguaianolides from *Artemisia anomala* (Asteraceae). *Biochem. Syst. Ecol.* 38 431–434. 10.1016/j.bse.2010.01.015

[B47] Zhou J. Du G. Chen J. (2014). Novel fermentation processes for manufacturing plant natural products. *Curr. Opin. Biotechnol.* 25 17–23. 10.1016/j.copbio.2013.08.009 24484876

