# Supplementary material for: Interleukin-8 Release Inhibitors Generated by Fermentation of Artemisia princeps Pampanini Herb Extract With Lactobacillus plantarum SN13T
Source: Front Microbiol. 2020 Jun 3;11:1159. doi: 10.3389/fmicb.2020.01159 (PMC7283739; doi:10.3389/fmicb.2020.01159)
Supplement: Supplementary file 1 [file Data_Sheet_1.PDF]

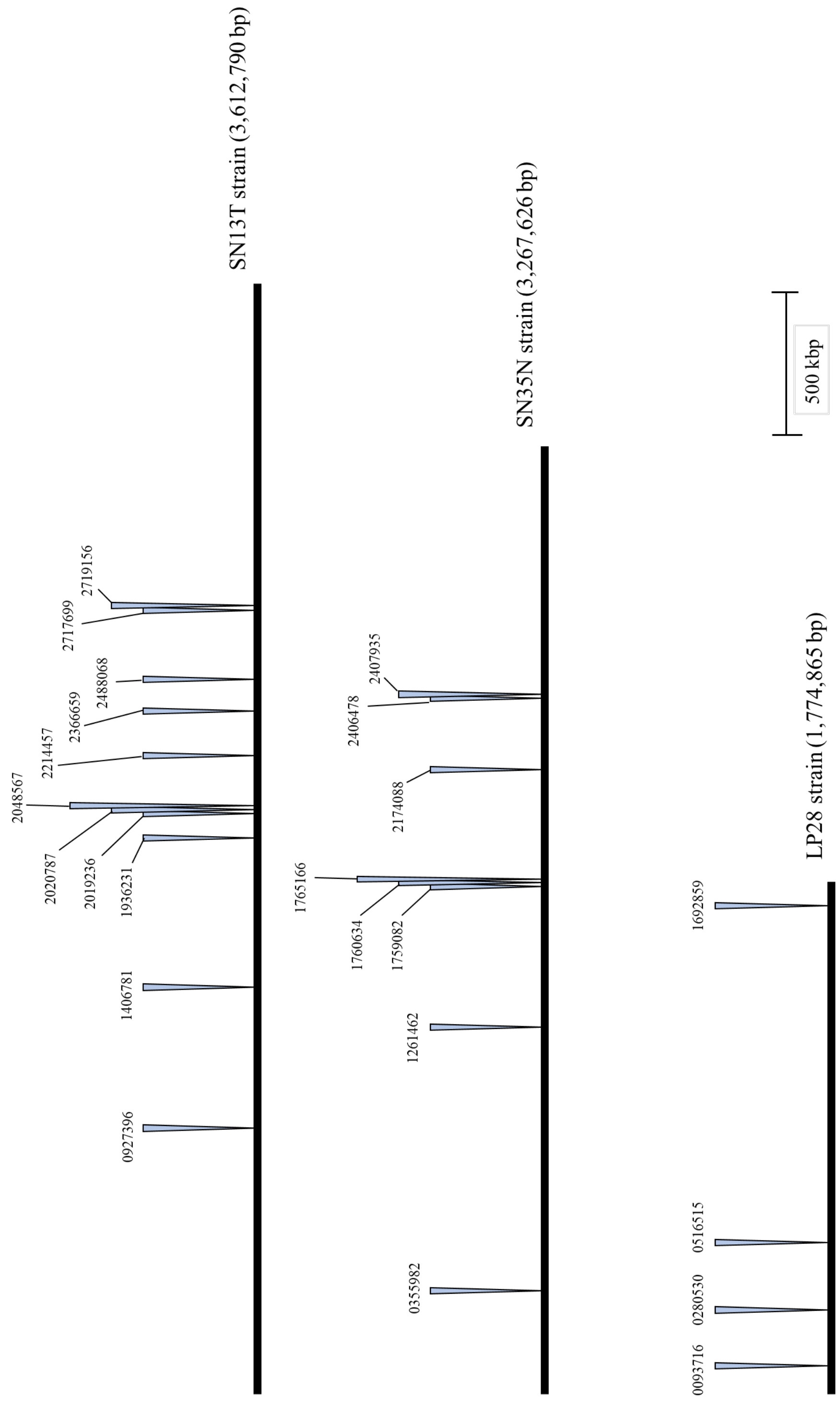

Figure S1. Putative β-glucosidase-encoding gene locus on the chromosome of each LAB strain. The wedges and numbers indicate the gene position on the chromosome.

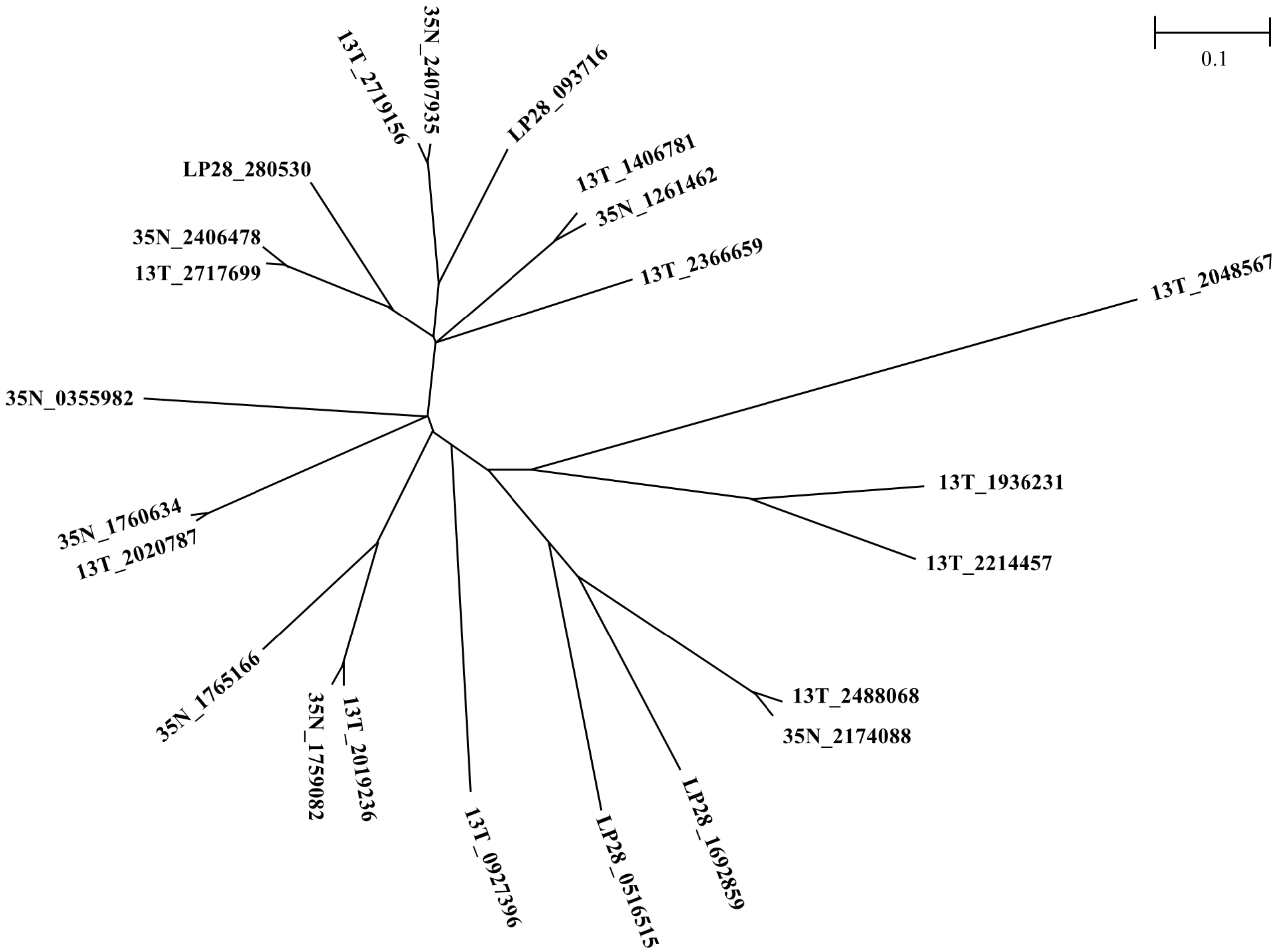

Figure S2

Phylogenetic tree on the β-glucosidase. The phylogenetic tree was drawn with the ClustalW program in Molecular Evolutionary Genetics Analysis (MEGA) software ver. 6.0 using the unweighted pair group method with arithmetic (UPGMA). The top horizontal bars show a distance of 0.1 substitutions per site.
